# Author Index for Volume 98

**DOI:** 10.1038/sj.bjc.6604446

**Published:** 2008-06-10

Abdelnoor, M 179

Abdullaev, ZK 571, 676

Abdulrahman, M 496

Abedini, MR 803

Abel, P 697

Abeliovich, D 2006

Abernethy, AP 294

Abnet, CC 1857

Adami, H-O 660

Adamo, B 1916

Adamo, V 1916

Adebamowo, CA 992

Ademuyiwa, F 992

Adenipekun, A 992

Adiguzel, Z 1675

Adlercreutz, H 636

Adrien, L 466

Aebi, S 25

Aghcheli, K 1857

Aguggini, S 1753

Ahmed, N 1415

Ahsan, H 992

Aikou, T 1389

Akaike, H 148

Akang, EE 992

Akashi, Y 749

Akiba, S 652

Akil, N 508

Akimoto, S 596, 2013

Akinmade, D 1132

Akiyama, Y 824

Akslen, LA 1662

Al Moustafa, A-E 508

Alam, SM 845

Albanell, J 1500

Albrektsen, G 189

Aleman, A 466

Allal, BC 120

Allegrini, G 1312, 1619

Allen, K 380

Allen, NE 1574

Allevi, G 1753

Allgaier, C 1525

Allgood, PC 206

Allonca, E 1274

Almassi, N 1244

Almonte, M 1704

Alsbeih, G 1845

Alvarez, E 1966

Alvord, EC Jr 113

Ambudkar, SV 1515

Amelsberg, A 80

Ammerlaan, ACJ 474

Amoroso, D 558

Anderson, E 840

Anderson, J 244

Anderson, JR 888

Anderson, LA 161

Anderson, R 1166

Andersson, A 1001

Andersson, M 728

André, T 875

Andreola, F 1675

Andreou, P 529

Andreuccetti, M 558

Ang, JE 1029

Angeli, A 1753

Angerson, WJ 888

Ansell, W 22

Ansink, AC 1342

Anton-Culver, H 1457, 2015

Antonini, N 137

Antoniou, AC 1457, 2015

Antonuzzo, A 558

Anttila, A 641

Aparicio, T 875

Appels, NMGM 1951

Appleby, PN 1574

Ararou, A 474

Archibeque, I 1059

Ardanaz, E 1574

Ardizzoni, A 71, 143

Arkenau, H-T 1029

Arnold, B 1508

Arnold, GE 1059

Artnik, B 1012

Arveux, P 217

Ashraf, SQ 1217

Aslett, H 529

Asselain, B 870

Åström, P 766

Awada, A 1500

Awasthy, B 1327

Axelsson, O 1285

Ayaru, L 1548 

Baade, PD 171

Baandrup, U 1292

Baas, F 474

Baba, H 974

Baba, Y 974

Baccaïni, B 225

Badie, C 1845

Bae, J-M 708, 1305

Bae, SH 542

Baek, JH 542

Bagby, GC 1653

Bagnardi, V 1745

Bahmanyar, S 1295

Bahrami, H 1443

Bai, T 803

Baik, I 660

Bailey, A 1704

Baker, E 1562

Baker, SD 1630

Bakker, MAden 1731

Bale, AE 282

Balin-Gauthier, D 120

Balteskard, L 1264

Bancroft, E 502

Bandstra, B 1608

Bang, S 881

Bang, Y-J 1118

Bao, L 1839

Barabutis, N 1790

Barak, V 77

Baras, M 2006

Barathova, M 129

Barbachanno, Y 502

Barletta, MT 1312

Barrett-Lee, P 34

Bartlett, J 502

Bartlett, JMS 1094

Barton, CA 1085, 1880

Baselga, J 1500

Baslé, MF 809

Basser, R 25

Bataille, B 1830

Bataille, F-R 1993

Bates, DO 1250, 1366

Bates, SE 1515

Batra, SK 1540, 1675

Batuman, OA 776

Baty, F 154

Bauer, A 1525

Bauer, HCF 434

Baylin, SB 1147

Bazley, L 380

Beck, PA 282

Becker, K-F 489

Beckers, T 604

Beesley, J 282

Beesley, S 1894

Beiderbeck, A 1870

Beijnen, JH 1320, 1951

Bekkers, RLM 165

Belbin, TJ 466

Bellec, S 225

Belobrajdic, DP 1593

Benjamin, K 1999

Berard, E 335

Berardi, E 328

Berardi, R 71

Berchuck, A 282

Beretta, GD 71

Berg, J 840

Bergkvist, L 636

Berglund, G 1574

Berkhof, J 646

Bermejo-Rodríguez, C 480

Bernhard, J 25

Bernstein, JL 728

Bernstein, L 728

Beroukhim, R 1662

Berruti, A 1753

Berry, CA 1696

Berry, DA 1729

Bertelsen, L 728

Berton-Rigaud, D 1993

Bevan, HS 1366

Bezjak, A 1769

Bhattacharyya, M 289

Bhopal, R 2011

Bianchi, M 263

Bianco, R 923

Biankin, AV 537

Bicknell, D 1217

Bielawska, A 98

Bielawski, J 98

Bieniek, E 1431

Bieri, G 1204

Bihl, MP 154

Bilim, V 941

Bingham, S 1574

Biollaz, J 1633

Biran, H 77

Birch, JM 1125

Birrer, MJ 915

Bischoff, H 1608

Bismar, TA 508

Bissonnette, RP 1380

Biswas, A 1210

Björkholm, M 1001

Black, R 840

Bladström, A 1876

Blagden, SP 894

Blanc, J-L 1830

Blechschmidt, K 489

Bliss, JM 34

Bloomfield, D 1894

Blot, W 232

Blouin, S 809

Blum, C 1999

Bocci, G 1312, 1619

Bodmer, WF 1217

Boeing, H 1574

Boffetta, P 1295, 1857

Bohnenkamp, H 784

Boice, JD 728

Bolijn, MJ 1951

Bollet, MA 734

Bonardi, S 1753

Boni, JP 1797

Bonneau, D 1830

Bonnetain, F 217

Bonomi, P 1608

Bopp, M 1012

Bordi, C 143

Borg, A 1457, 2015

Borrell, C 1012

Børresen-Dale, A-L 728

Borrow, R 1595

Bosch, FX 15

Boterberg, T 1723

Bottini, A 1753

Bouffler, S 1845

Boukovinas, I 1710

Boult, JKR 1985

Bourbouloux, E 1993

Bover, I 1608

Bowen, C 894

Bowen, R 1483, 2012

Bowen, RL 277

Boyle, PJ 1006

Bradshaw, N 840

Brand, RE 1540

Brändle, M 300

Braunstein, MJ 776

Breda, E 1910

Bressi, C 1046

Bridgewater, J 716, 1425

Britten, CD 523

Britton, PD 1741

Brock, A 2011

Brockhaus, B 900

Broderick, P 1434

Brown, CH 1741

Brown, JM 39

Brown, M 496

Brown, PH 1380

Brown, RF 1508

Brown, SR 39

Brown, W 1059

Brugnatelli, S 328

Bruland, O 1264

Brulport, M 1525

Brunton, V 1274

Bubendorf, L 154

Bucher, N 523

Buclin, T 1633

Bueche, D 300

Bueno-de-Mesquita, HB 1574

Bugat, R 120

Bui, S 143

Bukawa, H 1357

Bulkmans, NWJ 646

Burchell, J 784

Burger, H 80

Buring, JE 989

Burkes, RL 1769

Burkey, BB 619

Burkhard, R 1204

Burnett, ME 776

Burns, J 1797

Bushell, M 1696

Busse, L 1059

Butori, C 956

Butow, PN 1508

Byrnes, GB 1475 

Caers, J 1966

Cairney, CJ 1467

Calcagno, AM 1515

Caldwell, GM 1437

Camisa, R 143

Campana, F 734

Campanini, N 143

Campbell, J 840

Campbell, L 931

Campbell, MJ 1985

Campbell, NC 60

Campbell, O 992

Campion, L 1993

Campone, M 1993

Canal, P 120

Canel, M 1274

Canney, PA 1500

Cao, J-M 863

Capanu, M 728

Caplen, NJ 1515

Caraway, H 1147

Carbone, A 1910

Cardillo, A 1745

Cardoso, F 1500

Carlini, B 263

Carnaghan, H 840

Carpenter, B 426

Carpenter, R 289

Carrière, P 171

Carter, SL 1662

Carty, K 1774

Casale, F 263

Cascinu, S 71, 143

Cassidy, A 270

Castell, F 1597

Castellsagué, X 15

Castiglione-Gertsch, M 25

Catalano, V 71

Cazaux, C 120

Ceccon, G 143

Cemazar, M 388

Cerhan, JR 161

Cerny, T 300

Cerri, E 1312

Cetnarkyj, R 840

Cevales, D 1046

Chacko, RT 1327

Chae, YS 542

Chakraborty, S 1540

Chan, JK 1191

Chan, K 1951

Chang, HM 316

Chanock, S 282

Chaplain, G 217

Chappard, D 809

Chatterjee, N 45

Chechlinska, M 512

Cheetham, S 1810

Chelmow, DP 660

Chen, J-H 54

Chen, L-m 1191

Chen, LW 611

Chen, S 1839

Chenevix-Trench, G 282

Cheng, KK 323

Chernukhin, I 571, 676

Cherqui, D 875

Cheung, MK 1191

Chewning, KJ 1515

Chiara, M-D 1274

Chiou, J-M 54

Cho, Y 1258

Choi, IJ 1305

Chou, P 1741

Christensen, J 728

Christensen, S 1870

Christiani, DC 689

Christov, CP 981

Chu, T-Y 863

Chung, CH 619

Chung, DH 1118

Chung, HY 542

Chung, J 86

Chung, JB 881

Chung, WY 1141

Ciardiello, F 923

Cichowska, A 2011

Clancy, JL 1085, 1880

Clarke, N 496

Clarke, R 1023

Clarke, SJ 91

Classe, J-M 1993

Clavel, J 225

Clavien, P-A 98

Cleary, MP 370

Clynes, M 564, 1641

Cnattingius, S 1285

Coates, AS 25

Coelho, H 529

Coens, C 1452

Coer, A 388

Coleman, D 1176

Coleman, RE 1736

Colleoni, M 25, 1745

Colombo, N 720

Colonna, M 217

Colosetti, P 335

Colvin, EK 537

Compton, CC 450

Conaghan, PJ 1217

Concannon, P 728

Condemi, G 1916

Contreras-Hernández, I 1762

Cook, GJR 1597

Cook, MB 174

Cook, NR 989

Cooke, F 1141

Corazza, GR 328

Cordon-Cardo, C 466

Corrao, G 1046

Corrias, MV 263

Cosgrove, LJ 1593

Cosman, PH 537

Costa, G 1012

Coulson, P 1176

Coupé, VMH 646

Cox, RA 238

Cozen, W 161

Crafa, P 143

Cramer, DW 282

Cramp, M 1166

Creaney, J 1562

Crocicchio, F 71

Crook, T 1452

Crown, J 1641

Csajka, C 1633

Cummings, S 992

Cunningham, AP 1457, 2015

Currow, DC 294

Curto, M 256

Czerucka, D 335 

Dahl, C 728

Dahl, O 1264

Dahm, F 98

Daidone, M-G 1467

Daimon, T 1039

Dame, MK 1646

Damiano, V 923

Danesi, R 1312, 1619

Daniele, G 923

Danzon, A 217

Dar, MM 894

D’Arcy, V 571, 676

Darnel, AD 508

Dau, D 263

Dautel, P 106

Davey Smith, G 210

Davidson, R 840

Davies, A 22

Davies, D 380

Davies, MJ 1085, 1880

Dávila-Loaiza, G 1762

Davis, S 161

Dawar, R 1327

Dawsey, SM 1857

Day, NE 206

de Bock, GH 950

de Boer, AGEM 1342

de Bono, J 1029

de Bono, JS 894, 1951

de Gelder, V 1723

de Hullu, JA 165

de Micco, C 818

De Neve, W 1723

De Raeve, H 1966

de Reijke, TM 1342

De Ruyck, K 1723

De Rycke, Y 870

de Sanjosé, S 15

de Vito Barascud, A 154

de Vries, EGE 80

Dean, JP 245

Dean, ME 697

Deans, DAC 243, 443

Dearnaley, D 1852

Dearnaley, DP 1894

Debiec-Rychter, M 684, 1633

Deboosere, P 1012

Decosterd, LA 1633

DeFazio, A 1085, 1880

Deffebach, ME 1653

Deheragoda, M 1675

Del Tacca, M 1312, 1619

Delbaldo, C 875

Delord, J-P 120

Demmer, R 300

Dendale, R 734

Denkert, C 604

Dent, E 1508

Denton, J 1403

Dering, J 1076

Dervan, PA 1141

Desai, M 1704

Desotelle, JA 1244

Dey, A 4

Dhindsa, N 1327

Di Lullo, L 1916

Di Marsico, R 558

Di Paolo, A 1312, 1619

Dibben, S 1403

DiCioccio, R 282

Dico, ML 558

Diéras, V 1320

Dietel, M 604

Digel, W 1608

Dimitropoulou, P 1852

Dimoska, A 1508

Dindo, D 98

Dindyal, S 1482

Dinesen, J 1292

Ding, Y 1398

Dirix, LY 1500

Dixon, AR 1366

Do, YR 542

Doan, T 1197

Docquier, F 571, 676

Dodd, A 1999

Dogliotti, L 1753

Doherty, R 502

Doñate, F 776

Donde, B 1586, 1596

Doolan, P 1641

Dosaka-Akita, H 915

Dovio, A 1753

Downey, P 1182

Dowsett, M 39

Dravet, F 1993

Dreano, M 335

Drebber, U 627

Drescher, A 1959

Drummond, S 840

Duchosal, MA 1633

Duffy, S 1483, 2013

Duffy, SW 206, 270, 277, 1741

Dummer, R 1922

Duncan, R 426

Dunlop, J 840

Duthe, F 1830

Dziwura, S 1845 

Easton, D 502, 1852

Easton, DF 1457, 2015

Eastwood, AJ 697

Ebi, N 907

Eccles, DM 1457, 2015

Eden, TOB 1125

Edmiston, K 660

Edwards, J 1094

Edwards, K 931

Edwards, R 282

Edwards, S 1852

Eeles, R 502, 1852

Eeles, RA 2006

Eerola, H 1457, 2015

Egan, KM 1781

Eisenfeld, AJ 1608

Eley, HL 443

Eliassen, AH 240

Elliott, S 1059

Ellis, P 34

Ellstrøm-Engh, M 1582

Enblad, G 1001

Endou, H 742

Engeland, K 1525

Engels, EA 161

Engelsen, IB 1662

Erickson, RL 174

Eriguchi, M 399

Eskens, FALM 80

Eskild, A 1582

Eslick, GD 627

Esnaola, S 1012

Esteller, M 466, 1881

Evans, DG 1457, 2015

Evans, S 1852

Ewald, JA 1244

Eyfjord, JE 1457, 2015 

Fabbri, A 558

Fackenthal, J 992

Faithfull, S 1903

Falcone, A 558, 1312, 1619

Fanelli, G 1619

Farina, L 1046

Farrar, D 571, 676

Farrar, WL 756

Farshid, G 1182

Faulkner, L 263

Fearon, KCH 243, 443

Fedele, S 633

Fejzo, MS 1076

Feld, R 1769

Felicioni, I 1046

Feng, J 1398

Feng, Z 1006

Fenouille, N 335

Ferrandina, G 1910, 1916

Ferrari, P 1574

Ferraro, G 1916

Field, JK 270

Fielding, S 1434

Figg, WD 1630

Fincham, L 1903

Fink, BN 2

Finn, R 1076

Finnon, P 1845

Fioravanti, A 1312, 1619

Fischbacher, CM 2011

Fiscus, RR 1803

Fisher, C 502

Fisher, GJ 1646

Fitzpatrick, P 1141, 1759

Fléjou, J-F 587

Floore, A 1425

Fluge, O 1264

Flynn, B 1141

Fodde, R 1886

Forbes, JF 25

Fordyce, A 840

Fossati, R 720

Fostel, JM 1515

Foster, CS 502

Fourchotte, V 734

Fourquet, A 734, 870

Fox, E 1141

Frame, MC 1274

Franco-Lie, I 179

Franken, P 1886

Fraser, M 803, 1803

Fredericksen, ZS 282

Frederiksen, BL 668

Freemont, AJ 1403

Freitag, L 1608

French, LE 1922

Frenel, J-S 1993

Friis, S 232

Fritzsche, FR 604

Früh, M 689

Fruscio, R 720

Fryzek, J 232

Fuh, K 1191

Fujii, H 148

Fujii, S 1682

Fujikane, T 1147

Fujimori, T 1682

Fujimoto, J 845

Fujisawa, M 356

Fujita, N 580

Fujiwara, Y 1670

Fukui, H 1682

Fukui, Y 596, 2013

Fukuoka, J 907

Fukuoka, M 749, 907

Fukuyoshi, Y 410, 1536

Fumagalli, M 143

Furutani, K 1039

Fushimi, K 1357

Fuwa, N 1039 

Gabra, H 1774

Gabrielson, E 1147

Gallagher, CJ 289

Gallagher, E 1141

Gambarota, G 1784

Gambotti, L 870

Gammon, MD 2

Garaventa, A 263

Garbi, A 720

García-Campelo, R 1608

Garcia-Closas, M 282

Garduño-Espinosa, J 1762

Garofalo, S 923

Garrett-Mayer, E 1999

Garzón-Arango, M 1274

Gascon, P 1500

Gatzemeier, U 1608

Gayther, SA 282

Gebhard, S 1525

Gediya, LK 1234

Gehr-Swain, B 1852

Gekeler, V 604

Gelardi, T 923

Gelber, RD 25

Gemmell, LK 1094

Generali, D 1753

Gentil-Brevet, J 217

Gentle, D 496

Georgiev, P 98

Georgoulias, V 1710

Giangreco, A 380

Giatromanolaki, A 1975

Gibadulinova, A 129

Gibbons, B 840

Gibcus, JH 950

Gietema, JA 80

Gil, T 1500

Gilbert, DC 1894

Gilbert, J 619

Gill, PG 1182

Gillatt, D 1250

Gilmour, H 243

Ginther, C 1076

Giorgi, R 818

Giovanoli, P 1922

Girre, V 1320

Glass, AG 45

Glenn, WK 510

Gloss, BS 1085, 1880

Glynne-Jones, R 716

Gobbi, PG 328

Golanska, E 1431

Goldhirsch, A 25, 1745

Goldrick, AM 1141

Gollins, SW 1210

Gong, Y 596, 2013

González, CA 1574

Gonzalez, FJ 1630

Gonzalez, J 1540

González-Herrero, I 480

Goode, EL 282

Gore, M 1774

Gornet, JM 875

Gorouhi, F 1443

Gorski, B 1457, 2015

Goto, T 1068

Goubin, A 225

Goudie, D 840

Grabowska, AM 1696

Graham, A 1999

Granados-García, V 1762

Granström, C 199

Gratama, JW 1731

Gratwohl, A 852

Graubard, BI 174

Greaves, MF 1125

Green, AC 282

Green, JA 1452

Green, MD 25

Greenberg, DC 1741

Greenspan, E 587

Gregor, M 309

Gregory, H 840

Gridley, G 161

Griessinger, E 335

Griffiths, C 2011

Griffiths, DFR 931

Griffiths, EA 965

Grijota, M 1870

Grilli, B 154

Grimshaw, MJ 784

Gronwald, J 1457, 2015

Groot, KR 380

Grosclaude, P 217

Grossmann, ME 370

Gu, J 1716

Guan, H 1250

Guan, X 776

Guastalla, J-P 1774

Guevara, N 956

Guggenheim, M 1922

Guilhot, J 1830

Guldberg, P 728

Gumbleton, M 931

Guntinas-Lichius, O 627

Guo, R 596, 2013

Guy, M 1852 

Haba, R 1109

Hacker, NF 1085, 1880

Haglund, B 1285

Haile, RW 728

Hale, GA 854

Hale, M 1586, 1596

Hall, A 1852

Hallissey, MT 1985

Hallmans, G 1574

Hallor, KH 434

Hama, S 345

Hamaguchi, T 832

Hamburger, AW 1132

Hamburger, T 2006

Hamilton, W 323

Hampton, JM 1781

Han, KS 86

Han, W 1839

Handgretinger, R 854

Hankinson, SE 240, 1288

Hannun, YA 98

Haraguchi, N 1824

Harling, H 668

Harper, SJ 1250, 1366

Harris, AL 1753, 1951, 1975

Harris, JW 1630

Harrison, M 716, 1141

Hart, I 1483, 2012

Hart, IR 277

Hartge, P 161

Hartmann, JT 309

Hartmann, LC 282

Harvey, K 1348

Harving, H 232

Hashimoto-Tamaoki, T 1670

Hass, HG 309

Hassan, AB 1366

Hasumi, K 399

Hatashita, E 749

Hatfield, ARW 1548

Hatzidaki, D 1710

Haupt, R 263

Havet, K 956

Hawinkels, LJAC 1820

Hayashi, N 974

Hayashi, S 450

Haylock, B 1210

Hays, LE 1653

He, FR 611

He, Y 363

He, YC 363

Heching, N 77

Heerschap, A 1784

Hei, TK 1839

Heidenblad, M 434

Heijenbrok-Kal, MH 547

Heinävaara, S 641

Heinzelmann-Schwarz, VA 1085, 1880

Heist, RS 689

Heitzmann, F 1204

Hemminki, K 199, 997

Hémon, D 225

Henderson, MJ 1085, 1880

Hengstler, JG 1525

Henry, J-F 818

Henshall, SM 537, 1085, 1880

Heo, DS 1118

Hermes, M 1525

Hershman, DL 1197

Herzog, M 154

Hess, V 1204

Heuch, I 189

Hewson, P 1166

Hey, Y 1403

Hidaka, T 345

Higgins, CD 1929

Highnam, R 210

Higo, M 1357

Hilakivi-Clarke, L 1485

Hilakivi-Clarke, LA 1288

Hiley, V 39

Hilger, RA 1959

Hill, DL 792

Hill, J 1380

Hiraishi, H 1682

Hirakawa, K 514

Hirano, S 1258

Hirano, T 596, 2013

Hiraoka, N 418

Hirashima, K 974

Hirashima, T 907

Hirata, J 1068

Hirata, K 1147

Hiripi, E 997

Hirohashi, S 418

Hisada, T 742

Ho, KY 457

Hoare, SF 1467

Hochhaus, A 309

Hocking, B 1879

Hoctin-Boes, G 1951

Hoda, R 1999

Hodge, JP 894

Hodges, ZC 697

Hoeben, A 684

Hoffman, J-S 120

Hoffmann, TK 627

Hofheinz, R-D 309

Höfler, H 489

Hofman, P 335, 956

Hofman, V 956

Hogdall, E 282

Hogendoorn, PCW 434

Hollingsworth, MA 1540

Holloway, S 840

Holman, CDJ 168

Holotnakova, T 129

Holst, B 300

Hommes, DW 1820

Hong, S-K 708

Hong, SP 881

Hopper, JL 1457, 1475, 2015

Horger, MS 309

Horiike, S 580

Horn, LC 1525

Horvath-Puho, E 183

Horwich, A 1894

Houben, MPWA 474

Houlston, RS 1434

Hsieh, C-C 660

Hsu, C-S 863

Hu, Z 1327

Huang, C 1109

Huang, GS 611

Huang, K-F 863

Huang, Y-K 863

Huang, Z 457

Hubert, A 2006

Huddart, RA 1894

Hug, B 1797

Hughes, J 1696

Huisman, H 80

Huitema, ADR 1320

Hulikova, A 129

Hulsebos, TJM 474

Hunink, MGM 547

Huo, D 992

Hürny, C 25

Hurria, A 517

Hurst, NG 1594

Hurt, EM 756

Hussain, A 1125

Hyun, MS 542 

Iacopetta, B 1555

Iannotti, N 1608

Ichikawa, K 1682

Ichinokawa, K 1258

Ichinose, Y 907

Ieda’, N 720

IJszenga, M 434

Iles, SM 1210

Im, S-A 1118

Imai, H 742

Imai, K 1147

Imbert, V 335

Immervoll, H 1264

Imura, J 1682

Inada, H 596, 2013

Ingemann-Hansen, O 1292

Innocenti, F 558

Inokuchi, H 1039

Inoue, H 410, 1536, 1824

Intra, M 1745

Invernizzi, G 1046

Ioffe, OB 45

Irving, A 529

Isakov, L 1336

Ishida, T 1536

Ishigami, T 1357

Ishii, H 410

Ishikawa, E 399

Ishikawa, K 1258, 1824

Ishizuka, T 742

Islami, F 1443, 1857

Iso, H 1602

Isobe, H 693

Ito, H 1357

Ito, YM 1161

Itoh, T 1258

Itoi, T 941

Itti, E 875

Iversen, T 179

Iwasa, M 580

Iwasa, T 749

Iwasaki, M 418

Iyama, K-i 974

Izumi, H 345 

Jackson, S 1166

Jacobs, PA 1929

Jacobsen, BK 189

Jacobson, JS 1197

Jacoby, DB 1653

Jaehde, U 1959

Jahan, I 845

Jain, M 194, 1540

Jameson, C 502

Janes, SM 380, 855

Jansen, G 857

Jarm, T 388

Jarrard, DF 1244

Jasani, B 931

Jass, JR 450

Jedinak, A 1348

Jeffreys, M 210

Jeon, TJ 881

Jeon, YK 1118

Jeong, EG 1533

Jesien, E 1431

Jeziorska, M 1403

Jiang, E 1839

Jiang, S-W 1076

Jin, M 1555

Jit, M 1595

Jochum, W 98

Johannsson, O 1457, 2015

Johnsen, NF 1574

Johnson, KJ 1570

Johnson, L 34

Jonasch, E 1336

Jones, CE 1437

Jones, JL 277

Jones, L 529, 1053, 1483, 2012

Jönsson, G 434

Jørgensen, T 668

Joung, JY 86

Juarez, JC 776

Judson, I 1029

Julka, PK 1327

Jung, FJ 1922

Jung, K 604 

Kabat, GC 194

Kacevska, M 91

Kadivec, M 388

Kadouri, L 2006

Kadoya, M 1258

Kaira, K 742

Kaito, M 580

Kajiwara, T 832

Kajiwara, Y 345

Kalland, KH 1662

Kallioniemi, O-P 1457, 2015

Kamangar, F 1443, 1857

Kamimura, K 148

Kammeijer, M 1342

Kamohara, Y 1824

Kanai, Y 742

Kanazumi, N 1690

Kang, HJ 316

Kang, S 1646

Kang, Y-K 316

Kantola, S 766

Kapp, DS 1191

Karayan-Tapon, L 1830

Kasai, S 418

Kashii, T 907

Kassab, A 508

Kassapa, C 1574

Kassner, PD 1059

Katakami, N 907

Kathman, SJ 894

Kato, H 596, 2013

Kato, K 832, 1690

Kato, T 941

Kato, Y 1357

Katsuragi, K 514

Kaufmann, K 300

Kaukel, E 1608

Kawaguchi, Y 148

Kawakami, K 1555

Kawakami, T 596, 2013

Kawanishi, S 580

Kawasaki, BT 756

Kawasaki, J 1348

Kawata, T 1357

Kaye, S 1029, 1774

Ke, Y-M 863

Keith, WN 677, 1467

Kench, JG 537

Kennecke, H 1810

Kennedy, S 1641

Kerbel, RS 1312, 1619

Kerin, MJ 1141

Kervinen, V 766

Key, T 1852

Key, TJ 1574

Khan, OA 1614

Khandelwal, A 1234

Khatibian, M 1857

Khaw, K-T 1574

Khoo, V 1903

Kieler, H 1285

Kiemeney, L 1574

Kikuchi, J 915

Kikuchi, Y 652

Kim, BS 316

Kim, CG 1305

Kim, D-W 1118

Kim, EH 240

Kim, HK 1305

Kim, H-T 1380

Kim, J 1336

Kim, JG 542

Kim, JH 1118

Kim, MK 542

Kim, MS 1533

Kim, NK 1305

Kim, S 708

Kim, TW 316

Kim, T-Y 1118

Kim, YJ 1118

Kim, YT 1118

Kim, Y-W 708, 1305

King, A 1320

King, M 529

Kinoshita, I 915

Kinoshita, T 1690

Kirova, YM 734, 870

Kirven, K 1999

Kishida, T 496

Kita, G-X 571, 676

Kita, Y 410, 1536

Kitchener, HC 1704

Kjær, SK 282

Klarmann, GJ 756

Kleinerman, ES 1250

Klenova, E 571, 676

Kloft, C 900

Kluin, P 950

Klump, B 309

Klussmann, JP 627

Ko, F-S 54

Kobayashi, Y 580, 1161

Kodaira, T 1039

Koeberle, D 1204

Koifman, S 664

Koishi, K 1670

Kojika, M 596, 2013

Kondo, S 1258

Konecny, GE 1076

Kono, K 148

Kopacek, J 129

Koppenhöfer, U 309

Koppiker, CB 1327

Kordek, R 1431

Kornum, JB 1870

Korpi, JT 766

Kosuge, T 418

Kote-Jarai, Z 502, 2006

Kotnik, T 388

Koukourakis, ML 1975

Koutsopoulos, A 1710

Kouzu, Y 1357

Kovar, A 900

Kowalewska, M 512

Kraan, J 1731

Kranjc, S 388

Kriegner, A 816

Krishna, M 1999

Kristiansen, G 604

Kroll, ME 1006

Krude, T 981

Kubben, FJGM 1820

Kubota, T 1301

Kudoh, S 907

Kuester, K 900

Kuipers, EJ 547

Kulczycka, D 1431

Kumano, M 356

Kumar, R 1132

Kunst, AE 1012

Kupnicka, D 1431

Kurahara, H 1389

Kurahashi, T 356

Kurisu, K 345

Kusunoki, Y 1602

Kuwaki, K 1161

Kvåle, G 189

Kwon, K-Y 542 

Läärä, E 766

Labianca, R 71

Lacey, JV 45

Lacitignola, L 263

Lae, M 734

Lagadec, P 335

Lagarde, SM 1102

Lagerlöf, I 1001

Lagiou, P 660

Lahtinen, M 766

Lalloo, F 1457, 2015

Lambe, M 660, 1295

Lambeau, G 587

Lambertenghi-Deliliers, G 1046

Lamers, CBHW 1820

Lamers, CH 1731

Lamph, WW 1380

Lancashire, R 323

Lane, DP 4

Langagergaard, V 183

Langers, AMJ 1820

Langholz, B 45, 728

Langley, RE 697

Lapierre, F 1830

Larrañaga, N 1574

Larsen, C-J 1830

Latif, F 496

Lauraine, EP 1774

Lausch, E 1525

Lauss, M 816

Lawson, JS 510

Le Guludec, D 875

Le, NH 1886

Lebtahi, R 875

Leclerc, A 1012

Lee, C-S 537

Lee, I-M 989

Lee, JH 708, 1305

Lee, JS 1305

Lee, J-S 316

Lee, KH 86, 542

Lee, K-H 1118

Lee, KM 708

Lee, MK 708

Lee, SH 1533

Lee, S-H 1118

Lee, WJ 881

Lee, WS 542

Leen, R 1226

Leenders, W 1250, 1784

Leighl, NB 1769

Leister, C 1797

Lele, SM 1540

Lemos, C 857

Lepage, D 1966

Leung, EL 1803

Leung, W 854

Levillain, P 1830

Levine, E 1210, 1845

Levitt, NC 1614

Lewison, G 1944

Leyvraz, S 1633

Li, L 1258

Li, M 619

Li, Y 1380

Li, YF 611

Liang, Y 564

Liao, C-Y 863

Liberski, PP 1431

Lichtman, SM 517

Lidang, M 1292

Liddle, C 91

Lightfoot, TJ 1125

Liloglou, T 270

Lim, SL 1452

Lin, B 1327

Lindeman, GJ 537

Lindemann, K 1582

Linderoth, J 1001

Linet, MS 161

Ling, CQ 363

Ling, Y 1398

Linseisen, J 1574

Linton, KM 1403

Lissoni, AA 720

Lissowska, J 282

Liu, B 1398

Liu, D 1109

Liu, G 689

Liu, HX 611

Liu, Q 660

Liu, SJ 363

Liu, Y 1398

Lo, S-S 54

Lobanenkov, V 571, 676

Loebinger, MR 380

Loh, A 1675

London, R 91

Longmuir, M 840

Lopez-Otin, C 766

Lopez-Serra, L 466, 1881

Lophatananon, A 1852

Lorenzo Bermejo, J 997

Lorusso, D 1916

Lorusso, V 1916

Losardo, PL 143

Loupakis, F 1312

Loussouarn, D 1993

Low, HP 660

Lu, C 1716

Lu, C-H 863

Lubinski, J 1457, 2015

Lugli, A 450

Lui, W-Y 54

Luinetti, O 328

Luini, A 1745

Lundberg, O 1012

Luo, W-m 1132

Lupatsch, F 1525

Lüpfert, C 900

Lutz, TA 300

Lynch, CF 728

Lynch, TJ 689 

Ma, D 1327

Ma, N 580

Maass, C 1784

Macdonald, S 60

Mackenbach, JP 1012

Macleod, U 60

Maeda, J 596, 2013

Maeda, S 1389

Maeder, MT 300

Maemura, K 1389

Magnusson, S 1876

Maher, ER 496

Main, LC 426

Makela, S 1485

Mäklin, H 766

Malekzadeh, R 1857

Malmer, B 1001

Malone, KE 728

Mandahl, N 434

Manenti, S 1046

Mangioni, C 720

Manjer, J 1574

Mannelqvist, M 1662

Manoukian, S 1457, 2015

Manson, JE 989

Mantzoros, CS 1864

Manuia, MM 776

Marciano, R 923

Margison, GP 1614

Mari, E 71

Mari, M 335

Marjani, HA 1857

Markman, M 1021

Marshall, C 1494

Marshall, T 323

Martikainen, P 1012

Martin, SE 1515

Martinelli, E 1910

Martinelli, G 25

Martinelli, R 143

Martinez, VG 564

Masi, G 1312

Mason, C 529

Massuger, LFAG 165

Mastik, MF 950

Masuya, D 1109

Mataki, Y 1389

Mather, J 1704

Matsubara, J 832

Matsui, K 693

Matsumoto, T 1670

Matsumura, Y 1258

Matsuura, S 345

Matthews, GM 1437

Matull, WR 1675

Mavroudis, D 1710

Maxwell, GL 1288

Mazar, AP 776

Mc Cann, A 1141

Mc Cormack, O 1141

McBride, R 1197

McCall, P 1094

McClatchey, AI 256

McCrory, DC 294

Mcfaul, S 22

McGlynn, KA 174

McGrath, SM 965

McGuire, V 282

McKernan, M 888

McLeish, L 840

McMillan, DC 888

Medema, JP 1226

Mehta, JP 1641

Meignan, M 875

Meijer, CJLM 646

Meijnen, P 137

Melia, J 1176

Mellemkjær, L 728

Meloni, G 1046

Mendez, P 1710

Menei, P 1830

Menkema, L 950

Menu, E 1966

Menvielle, G 1012

Mertens, F 434

Merup, M 1001

Mesak, F 1810

Messerschmidt, J 1959

Meyer, A 106

Miccolis, IR 1046

Michael, M 1614

Michalak, S 1830

Michel, RP 450

Michels, KB 1288

Middleton, M 1951

Middleton, MR 894, 1614

Midgley, R 1614

Miedzybrodska, Z 840

Mihic-Probst, D 1922

Miklavcic, D 388

Milani, M 1753

Milano, G 1320

Milin, S 1830

Miller, AB 194

Miller, CJ 1403

Miller, DJ 1023

Miller, E 1595

Miller, ID 1327

Millward, MJ 1562

Milone, G 1046

Mimori, K 410, 1824

Min, HS 1118

Minamoto, T 1555

Mishra, R 1258

Mitas, M 1999

Mitchell, AJ 1934

Mitchell, E 60

Mitra, A 502

Mitsui, S 399

Miya, T 1034

Miyake, H 356

Miyamoto, M 1258

Miyanari, N 974

Mizukami, Y 148

Mizuno, NK 370

Modugno, F 282

Moles, DR 633

Molife, LR 894

Molitor, M 627

Mom, CH 80

Money-Kyrle, J 1894

Moniaux, N 1540

Montagna, E 1745

Montravers, F 875

Moody, J 1845

Mooney, T 1759

Moore, J 1555

Moratti, R 328

Mori, K 693

Mori, M 410, 742, 1147, 1536, 1824

Mori, T 1690

Morishima, K 345

Morishita, Y 399

Moriwaki, T 832

Morris, MR 496

Mortensen, NJM 1217

Mortimer, P 1614

Morton, DG 1437

Morton, LM 161

Mosnier, J-F 1993

Moss, S 1176, 1704

Mössner, J 1525

Motoyama, K 410

Motoyama, T 941

Mould-Quevedo, JF 1762

Mounier, CM 587

Moysich, K 282

Muir, K 1852

Mukai, K 596, 2013

Mukherjee, R 1094

Murad, S 529

Murali, R 1880

Murch, AR 1562

Murday, V 840

Murphy, B 619

Murphy, MFG 171

Murphy, NC 537

Murray, GI 426

Musgrove, EA 537

Muto, A 941

Mueller, GA 1525

Myint, S 1210

Myklebust, MP 1264

Myles, JP 270

Myles, P 1852 

Naccarato, AG 1619

Nag, S 1327

Nagai, Y 974

Nagaoka, A 941

Nair, A 1327

Nair, S 1327

Nakagawa, K 693, 749

Nakahara, R 1039

Nakajima, T 742

Nakajima, TE 832

Nakamura, S 1161

Nakamura, T 1039

Nakano, J 1109

Nakano, Y 1670

Nakao, A 1690

Nakashima, D 1357

Nakayama, T 1602

Naraynsingh, V 1482

Narod, SA 1457, 2015

Nasrollahzadeh, D 1857

Natsugoe, S 1389

Navani, N 380, 855

Nawa, K 596, 2013

Nawrot, MP 335

Nazareth, I 529

Neale, RE 171

Negoro, S 907

Negri, F 263

Negri, FV 143

Nehls, O 309

Nejim, A 39

Nelson, PS 245

Nemirovsky, I 77

Ness, RB 282

Netterville, JL 619

Neuweiler, J 1204

Nevanlinna, H 1457, 2015

Newcomb, PA 1781

Ngan, S 1

Nguyen, KCQ 1059

Nicholson, D 840

Niemann, I 1292

Niesporek, S 604

Nishikawa, N 1147

Nishimura, M 915

Nishimura, T 907

Nishina, T 832

Nishioka, NS 689

Nishiwaki, Y 693

Nisman, B 77

Njar, VCO 1234

Nkhata, KJ 370

Nocito, A 98

Noehammer, C 816

Noh, J-H 708

Nojima, M 1147

Nokihara, H 693

Noll, T 784

Noller, KL 660

Nomoto, S 1690

Nørgaard, M 183

Nørgård, B 183

Norman, G 697

Nouraie, M 1857

Nouso, K 1161

Nowak, AK 1562

Nowak, R 512

Nwosu, AC 1053

Nyrén, O 1295 

O’Brien, L 1852

O’Brien, M 1608

O’Brien, PM 1085, 1880

O’Connor, R 564

O’Driscoll, L 1641

Oettle, H 309

Ogata, A 399

Ogiwara, A 596, 2013

Ogundiran, TO 992

Oh, DS 1327

Oh, D-Y 1118

Oh, ST 316

Ohashi, Y 1161

Ohira, T 596, 2013

Ohmura, T 1147

Ohradanova, A 129

Ohta, M 410

Ohtsu, A 1034

Ojima, H 418

Oka, T 596, 832, 1555, 2013

Okamoto, I 749, 907

Okayama, Y 832

Okoturo, O 1548

Olah, E 1457, 2015

Oldham, FB 1608

Oliver, T 22

Olmos, D 1029

Olopade, OI 992

Olsen, JH 232, 728

Olson, SB 1653

Olsson, H 1457, 1876, 2015

Olver, I 1614

Ono, K 749

Ooyama, A 1555

Oriuchi, N 742

Orlandi, P 1312, 1619

Orsini, N 1864

Osann, K 1191

Osler, M 668

Otsubo, T 824

Otte, AP 1662

Ouellette, M 1540

Overvad, K 1574

Owen, D 1810

Øyan, AM 1662 

Pack, S 571, 676

Page, RD 270

Paglia, A 1916

Pagliaro, LC 1336

Palli, D 1574

Panis, Y 875

Pankow, JF 1653

Papazisis, K 784

Paraskeva, C 1366

Park, JY 881

Park, KU 542

Park, M-S 881

Park, SR 1305

Park, SW 881

Parkkila, S 129

Parmar, MKB 697

Parodi, S 263

Parshad, R 1327

Pasini, B 2015

Passini, B 1457

Pastorek, J 129

Pastorekova, S 129

Patel, M 1586, 1596

Patterson, KI 1085, 1880

Paul, J 1774

Paulussen, M 852

Pavanetto, M 1046

Payne, D 1769

Payne, M 894

Paz-Ares, L 1608

Pearce, CL 282

Pearson, P 840

Pebody, R 1595

Pedersen, L 1870

Penegar, S 1434

Peock, S 502

Pepper, SD 1403

Pereira, SP 1548, 1675

Peretz, T 77, 2006

Perez, C 1336

Pérez-Caro, M 480

Perone, P 1646

Perou, CM 1327

Perren, TJ 39

Perrin, RM 1366

Pescador, L 1046

Peters, GJ 857

Peters, TJ 323

Peterse, JL 137

Petersen, JA 1762

Petersen, LK 1292

Peto, J 1457, 1704, 2015

Petrillo, M 1911

Peyron, JF 335

Pfeifer, S 1285

Pfüller, U 106

Pharoah, PDP 282, 1457, 2015

Phatak, A 1593

Piaskowski, S 1431

Piccart, MJ 1500

Picco, G 784

Pieck, AC 1959

Pierga, J-Y 734, 870

Pieterse, S 1182

Pignata, S 1910

Pike, MC 9, 282

Pillaire, M-J 120

Pioud-Martigny, R 1993

Pirilä, E 766

Piris, MA 480

Pischon, T 1574

Pisconti, S 1916

Pittman, AM 1434

Planting, AST 80

Platten, MA 1562

Plymate, SR 250

Pocock, R 1852

Poddigue, M 1046

Podkrajsek, M 388

Podratz, KC 1076

Pombo-de-Oliveira, MS 664

Porcellana, M 1046

Pore, N 571, 676

Porteous, M 840

Porter, SR 633

Poston, GJ 1053

Poulsen, AH 232

Pour, PM 1540

Pourshams, A 1857

Powell, CB 1191

Powers, S 1059

Powles, T 22

Prawitt, D 1525

Prendiville, J 1608

Preuss, SF 627

Price, KN 25

Price, PM 965

Prichard, L 380

Priolo, D 1916

Prisco, M 1910

Pritchard, DM 1053

Pritchard, SA 965

Propper, D 716

Protheroe, AS 894

Pucci, F 71, 143

Purushotham, AD 1741

Puumala, SE 1570 

Qian, X 1398

Qiu, Y 1366

Quennell, A 1903

Quinn, DI 1085, 1880

Quinn, MA 1415

Quirós, JR 1574

Qureshi, U 1675 

Radford, JA 1403

Radice, P 1457, 2015

Raffy, C 1845

Raffy, S 1366

Rahmeh, M 1076

Rai, S 571, 676

Ramdass, MJ 1482

Ramsay, A 529

Ramu, N 77

Ramus, SJ 282

Rani, S 1641

Ranson, M 1614

Rapoport, EA 242

Rashid, M 1548

Ray, A 370

Rayburn, ER 792

Ream, E 1903

Reed, MJ 250

Regidor, E 1012

Reid, AHM 894

Reis, M 840

Reiterer, P 1608

Rennel, ES 1250, 1366

Riboli, E 1574

Richardson, A 529, 1903

Richel, DJ 1102

Richesson, DA 45

Richmond, E 1320

Rieske, P 1431

Rigas, B 1157

Rigoard, P 1830

Riley, C 1415

Rinaldi, S 1574

Ripert, M 225

Risch, HA 282, 1457, 2015

Rischewski, J 852

Ristimäki, A 766

Ritchie, G 697

Riviere, A 1608

Roberts, C 1704

Robertson, G 91

Robinson, BWS 1562

Robsahm, TE 179

Rochaix, P 120

Roddam, AW 1574

Rodermond, HM 1226

Rodriguez, J 1745

Rogers, G 1166

Rogers, N 1059

Rohan, TE 194

Rohrmann, S 1574

Romano, M 1999

Ronnett, BM 45

Rosa, R 923

Rosell, R 1398, 1710

Rosenberg, DW 587

Rosenberg, I 1020

Röske, A 604

Ross, H 1608

Ross, JA 243

Rosselet, A 1633

Rossello, R 1916

Rostomily, RC 113

Rotmensz, N 1745

Rougier, P 875

Rousseau, D 335

Rousson, V 1922

Rubertone, MV 174

Rudant, J 225

Ruff, P 1586, 1596

Rufle, A 154

Ruhstaller, T 1204

Rush, BB 45

Rust, C 1204

Rustin, G 1774

Ruszkiewicz, A 1555

Rutgers, EJT 137

Ryan, D 289, 1483, 2012

Ryan, DA 277

Ryder, S 1166

Rylander-Rudqvist, T 636

Ryoo, HM 542

Ryu, KW 708, 1305

Ryu, M-H 316 

Saarinen, NM 1485

Sagan, C 1993

Saijo, N 693

Sainsbury, JR 39

Saito, K 1357

Saito, T 345

Saji, S 636

Salcido, CD 1515

Salinas-Escudero, G 1762

Salmon, R 734

Salo, T 766

Salvagni, S 71

Salvesen, HB 1662

Samsa, GP 294

Sanchez, JJ 1398

Sánchez, M-J 1574

Sánchez-Beato, M 480

Sanchez-Carbayo, M 466

Sánchez-García, I 480

Santini, J 956

Sargent, A 1704

Sarkeala, T 641

Sasaki, Y 1034

Sasako, M 1670

Sassen, S 489

Sasson, AR 1540

Sato, E 845

Sato, M 1389

Satoh, T 749, 907, 1034

Saunders, DN 1085, 1880

Saunders, E 1403

Savarese, A 1910

Savarese, TM 660

Savchenko, V 818

Savic, S 154

Savignoni, A 734

Sawa, T 907

Scambia, G 1910, 1916

Scarano, E 1745

Scarlett, CJ 537

Scartozzi, M 71

Schally, AV 1790

Schellens, JHM 1320, 1951

Schepelmann, S 674

Scheulen, ME 1959

Schiffer, IB 1525

Schildkraut, J 282

Schmalfeldt, B 489

Schmidt, M 1525

Schoemaker, MJ 1929

Schöffski, P 684

Schormann, W 1525

Schüler, Y 1250

Schumacher, U 106

Schumann, A 1525

Schuster, T 489

Schuuring, E 950

Scolyer, RA 1085, 1880

Scott, D 1845

Scotti, L 1046

Scuderi, F 263

Scurr, L 1085, 1880

Scurry, JP 1085, 1880

Sebag, F 818

Secades, P 1274

Secerov, A 388

Seebaran, A 894

Segal, A 1562

Segara, D 537

Sekikawa, A 1682

Sekine, I 693

Sellers, TA 282

Selva, E 956

Sementa, AR 263

Semnani, S 1857

Semrau, R 627

Sender, M 1001

Sentjurc, M 388

Seo, HK 86

Seong, J 881

Serrels, A 1274

Sersa, G 388

Severson, RK 161

Shakeri, R 1857

Shamash, J 22

Sharma, R 91

Sharp, D 323

Shaw, DE 776

Shay, JW 677

Shen, Q 1380

Shepherd, FA 689, 1769

Sherman, ME 45

Shibata, K 907

Shibata, T 418

Shichinohe, T 1258

Shimada, Y 832, 1034

Shimizu, E 907

Shimizu, K 742

Shimizu, Y 915

Shimoda, T 832

Shin, JY 1191

Shinchi, H 1389

Shinohara, T 1258

Shinomura, Y 1147

Shirao, K 832, 1034

Shoji, Y 1258

Shostrom, VK 1540

Shrestha, P 345

Shrout, J 1999

Shyr, Y 619

Siebers, AG 165

Siegel-Lakhai, WS 1320

Sier, CFM 1820

Siersema, PD 547

Sigal-Zafrani, B 734

Silva I dos, S 633

Silva, A 1762

Simpson, C 380

Sinard, R 619

Sinclair, AM 1059

Singer, JW 1608

Sitas, F 1586, 1596

Skarstein, A 1264

Skipworth, RJE 443

Slamon, DJ 1076

Slater, S 840

Slebos, RJC 619

Sleijfer, S 1731

Slimani, N 1574

Sliva, D 1348

Slivova, V 1348

Smart, M 571, 676

Smith, AN 1085, 1880

Smith, ELP 776

Smith, K 840

Smith, NF 1630

Smith, P 1452

Smyth, E 840

Snadden, L 840

Snijders, PJF 646

Sobhani, I 875

Sobrero, A 71

Sogabe, Y 1147

Sohn, H-J 316

Sohn, SK 542

Sohn, T-S 708

Soldan, K 1595

Soler, JT 1570

Song, H 282

Song, HS 542

Song, SY 881

Sonoda, T 1147

Soon, Y 1437

Sørensen, HT 183, 232, 1870

Sorsa, T 766

Sotoudeh, M 1857

Souglakos, J 1710

Southey, MC 1457, 2015

Sparreboom, A 1630

Spector, LG 1570

Speel, EJM 627

Speight, PM 633

Spelten, ER 1342

Spitz, MR 1716

Sprangers, MAG 1342

Sprenger, CC 250

Springer, CJ 674

Srivastava, P 1336

Staaf, J 434

Stålberg, K 1285

Standop, J 1540

Stathopoulos, E 1710

Stattin, P 1574

Stawski, R 1431

Stebbing, J 716

Steck, SE 2

Steel, M 840

Stefansson, IM 1662

Stein, K 1166

Stein, L 1586, 1596

Steinert, H 1922

Stenner, M 627

Stephan, C 604

Stephens, TC 1951

Stern, M 852

Sterrett, G 1562

Stewart, DJ 1716

Stewart, S 515

Stieler, J 309

Stiller, CA 1006

Stirbu, I 1012

Stirling, D 840

Stoeber, K 1548

Stopfer, P 80

Stovall, M 728

Stram, DO 282

Strand, BH 1012

Strasser, F 300

Strijbos, MH 1731

Strohsnitter, WC 660

Strumberg, D 1959

Stuart, RC 888

Su, L 689

Suarez, C 1274

Suga, Y 596, 2013

Sugai, H 148

Sugimoto, H 1690

Sugimoto, R 580

Sugiono, M 1250

Sugiyama, K 345

Sullivan, K 1434

Sullivan, R 1944

Sum, EYM 537

Sumitomo, S 1109

Sun, A 1769

Sun, SH 363

Sun, Y 1157

Sun, YM 363

Sunaga, N 742

Sundar, S 238

Sundquist, J 199, 997

Sung, SW 1118

Supuran, CT 129

Susanto, J 537

Susnerwala, S 1210

Sutherland, RL 537, 1085, 1880

Suzuki, H 1147

Suzuki, M 749

Suzuki, R 636

Suzuki, T 1602

Svanholm, H 1292

Swallow, DM 1675

Swanson, KR 113

Swerdlow, AJ 1929

Swindell, R 1210, 1403

Sydes, MR 697

Syed, N 1452

Syrjakoski, K 1457, 2015

Szlosarek, P 1452

Szuhai, K 434

Szulc, ZM 98

Szybka, M 1431 

Tachibana, H 1039

Tagliabue, G 1574

Tagliaferri, P 71

Tahara, M 1034

Tai, IT 1810

Takacova, M 129

Takamoto, S 399

Takano, M 1068

Takao, S 1389

Takebayashi, T 652

Takeda, K 693

Takeda, S 1690

Takei, Y 580

Taki, M 652

Talbot, JN 875

Talukder, AH 1132

Tamaya, T 845

Tamboli, P 1336

Tampellini, M 1753

Tamura, K 907

Tamura, T 693

Tanaka, F 1824

Tanaka, H 580

Tanaka, S 742

Tang, MJ 1810

Tang, N 1457, 2015

Tanière, P 1985

Tanigawara, Y 1034

Tannir, N 1336

Tanzawa, H 1357

Tao, L 1670

Tapia, C 154

Taron, M 1710

Tartarelli, G 558

Tattersall, MHN 1508, 1769

Tavelin, B 1001

Taylor, GM 1125

Taylor, J 1975

Taylor-Papadimitriou, J 784

Tchilian, E 1217

Tedoldi, S 1753

Teepen, JLJM 474

Teh, BT 496

Teh, M 457

Teh, SK 457

Telfer, C 426

ten Kate, FJW 1102

Terracciano, L 154

Terraciano, L 1204

Terry, KL 282

Thierens, H 1723

Thies, A 106

Thomas, DC 728

Thomas, G 766

Thomas, R 1494

Thomas, SB 756

Thompson Coon, J 1166

Thomson, C 1704

Thorpe, H 39

Thuerlimann, B 300

Thürlimann, B 25

Tian, DF 363

Tibaldi, C 558

Tibben, MM 1320

Tibrewal, S 380

Tichelli, A 852

Tijssen, CC 474

Timucin, C 776

Tiret, E 875

Tisdale, MJ 243, 443

Tjønneland, A 232, 1574

To, KKW 1515

Toepfer, M 1204

Toft, D 1076

Tokino, T 1147

Tomita, S 1682

Tomita, Y 941

Tomlinson, I 1434

Tomoda, T 1039

Tookman, A 529

Tormo, M-J 1574

Torri, V 720

Torrisi, R 1745

Torta, M 1753

Tortora, G 923

Toyoda, Y 1602

Toyota, M 1147

Travis, RC 1574

Trentham-Dietz, A 1781

Trichopoulos, D 660, 1574

Trichopoulou, A 1574

Tripaki, M 1710

Trivier, E 981

Trojan, J 309

Tronconi, C 328

Tryggvadottir, L 1457, 2015

Tsang, BK 803, 1803

Tschöp, M 300

Tselepis, C 1985

Tseng, C-C 9

Tsigani, T 1845

Tsuchikawa, T 1258

Tsuda, H 1068

Tsujimura, T 1670

Tu, SM 1336

Tucci, M 1753

Tumino, R 1574

Turner, A 1704

Turner, V 854

Tuynman, JB 1102

Twu, N-F 863

Tytherleigh, MG 1217 

Ueno, M 1109

Uhlemann, K 1525

Uitterhoeve, ALJ 1342

Urawa, N 580

Urban, MI 1586, 1596

Uronis, HE 294

Utsunomiya, T 1536

Uzawa, K 1357 

Väänänen, A 766

Vadivelu, S 619

Valentine, HR 965

Valentino, F 328

van As, NJ 1894

van Bree, C 1226

Van Camp, B 1966

van de Nieuwenhof, HP 165

van de Vijver, MJ 137

van der Burg, M 1452

van der Kogel, B 1784

van der Reijden, JJ 1820

van der Wal, JE 950

van Dijck, JAAM 165

van Dijk, FJH 1342

van Doorn, L 80

van Duijn, W 1820

Van Eijkeren, M 1723

van Ham, MAPC 165

van Kuilenburg, ABP 1226

van Laar, R 1425

van Lanschot, JJB 1102

Van Obberghen-Schilling, E 956

van Tellingen, O 1784

van Tongeren, M 270

Van Valckenborgh, E 1966

van Vliet, EPM 547

Vanderkerken, K 1966

Van’t Veer, L 1425

Varani, J 1646

Varey, AHR 1366

Varsier, N 652

Varticovski, L 1515

Vasey, PA 1774

Vasile, E 558

Vasist, LS 894

Vatten, LJ 1582

Vaylet, C 875

Velten, M 217

Venkatesan, N 1076

Verbeek, JHAM 1342

Verma, CS 4

Veronesi, P 1745

Verspaget, HW 1820

Verweij, J 80

Viacava, P 1619

Viale, G 1745

Vierlinger, K 816

Villasís-Keever, MA 1762

Vincent-Salomon, A 734

Vineis, P 1574

Vinjamuri, S 1053

Vinnicombe, S 289

Visne, I 816

Visvader, JE 537

von Moos, R 300, 1204

Von Pawel, J 1608

Voorhees, JJ 1646

Vora, J 1053

Vral, A 1723

Vult von Steyern, F 434

Vuong, T 450

Vyse, A 1595 

Wager, M 1830

Wain, JC 689

Waine, E 1250

Wake, K 652

Waldron, J 1769

Walker, L 1494

Wallace, M 1999

Wanders, J 1320

Wang, D 611

Wang, H 792

Wang, J 1839

Wang, JH 611

Wang, L 611

Wang, W 792

Wang, WQ 611

Wang, Y 1023

Wang, Z 611

Warner, E 1457, 2015

Warner, RL 1646

Warrack, R 1437

Warren, R 210

Warren, RML 206

Warri, A 1485

Warwick, J 206

Watanabe, G 1555

Watanabe, S 580, 652

Watson, AJ 1614

Watson, JEV 1059

Watson, SA 1696

Watt, C 840

Watts, CKW 1085, 1880

Waxman, J 1

Webb, E 1434

Webb, PM 282

Weber, G 1959

Weber, M 1586, 1596

Webster, B 1475

Webster, GJ 1548

Wei, J 1398

Weichert, W 604

Weinell, A 627

Weinhaeusel, A 816

Weishaupt, M 1525

Weissenborn, SJ 627

Welch, IM 965

Weller, D 60

Wendum, D 587

Wernli, KJ 1781

Wesseling, P 474, 1784

West, CML 965

Whang-Peng, J 54

Whitaker, NJ 510

White, VM 1475

Whiteman, DC 282

Whittemore, AS 282

Whyte, C 840

Widmer, N 1633

Wiesmann, KG 1959

Wigfield, SM 1975

Wigmore, SJ 243

Wild, SH 2011

Wilding, JL 1217

Wilhelm, C 1525

Wilkinson, R 1852

Willett, WC 240

Williams, DD 894

Williams, ED 1085, 1880

Williams, G 1548

Williams, M 1494

Willis, AE 1696

Wilson, CL 1403

Wilson, P 22

Wilson, R 1774

Wilson-Barnett, J 1903

Winston, A 1759

Winter, SC 1975

Winterhalder, R 1204

Wishart, GC 1741

Wittekindt, C 627

Wolff, MS 2

Wolk, A 636, 1864

Wollenschlaeger, A 1548

Wong, H 1452

Wood, C 1336

Woolard, J 1250

Wright, AF 1929

Wright, D 1166

Wright, JD 1197

Wu, AH 9, 282

Wu, C-H 863

Wu, C-P 1515

Wu, C-W 54

Wu, L 1839

Wu, X 1716 

Xie, L 1398

Xing, J 1716

Xu, A 1839

Xue, F 1288 

Yalniz, M 1540

Yamada, Y 749, 832, 1034

Yamagishi, H 1682

Yamaguchi, N 652

Yamamoto, K 1161

Yamanaka, K 356

Yamasaki, F 345

Yamase, T 399

Yamashita, A 399

Yamashita, K 410

Yamazaki, K 915

Yan, QG 611

Yanagie, H 399

Yanagihara, K 824

Yanagitani, N 742

Yang, G 1076

Yang, X 803

Yao, M 496

Yarbrough, WG 619

Yashiro, M 514

Yasmeen, A 508

Yates, JE 1653

Ye, W 1295, 1857

Yeo, W 25

Yeoh, KG 457

Ylipalosaari, M 766

Yokomise, H 1109

Yoo, NJ 1533

Yook, JH 316

Yoshida, N 974

Yoshida, T 749

Yoshikawa, D 418

Yoshikawa, R 1670

Yoshioka, T 1258

Yoshitake, N 1682

You, S-L 863

Young, D 840

Yousefzadeh, M 1999

Yu, L 1398

Yu, MC 9

Yu, W 542

Yu, Z 1839

Yuan, C-C 863

Yuasa, Y 824

Yun, YH 708

Yuuki, K 941 

Zaccheo, O 1366

Zaffaroni, N 1467

Zahrieh, D 25

Zakrzewska, M 1431

Zambon, A 1046

Zandvliet, AS 1320

Zannoni, G 1910

Zatovicova, M 129

Zawisza, D 1769

Zawlik, I 1431

Zendehdel, K 1295

Zeng, L 363

Zeng, Y 1085, 1880

Zhai, R 689

Zhang, JJ 25

Zhang, M 168

Zhang, R 792

Zhang, SM 989

Zhang, WP 611

Zhang, X 168

Zhang, Y 174, 1132, 1380

Zhao, G 1839

Zhao, X 168

Zhao, Y 792, 1398, 1839

Zheng, W 282, 457

Zhou, W 689

Zhu, L 1839

Zlobec, I 450

Zodrow, DM 1653

Zou, Z 1398

